# The Underlying Molecular Basis and Mechanisms of Venous Thrombosis in Patients with Osteomyelitis: A Data-Driven Analysis

**DOI:** 10.1155/2022/5672384

**Published:** 2022-06-06

**Authors:** Peisheng Chen, Yinhuan Liu, Xiaofeng Lin, Bin Yu, Bin Chen, Fengfei Lin

**Affiliations:** ^1^Department of Orthopaedics, Fuzhou Second Hospital of Xiamen University, School of Medicine, Xiamen University, Fuzhou 350007, China; ^2^Department of Orthopaedics, Fuzhou Second Hospital, The Third Clinical Medical College, Fujian Medical University, Fuzhou 350007, China; ^3^Fujian Provincial Clinical Medical Research Center for First Aid and Rehabilitation in Orthopaedic Trauma, Fuzhou Trauma Medical Center, Fuzhou 350007, China; ^4^Department of Laboratory Medicine, Fuzhou Second Hospital, Fuzhou 350007, China; ^5^Department of Endocrinology, Fuzhou Second Hospital, Fuzhou 350007, China; ^6^Division of Orthopaedics & Traumatology, Department of Orthopaedics, Nanfang Hospital, Southern Medical University, Guangzhou 510515, China

## Abstract

**Objective:**

Osteomyelitis (OM) is one of the most risky and challenging diseases. Emerging evidence indicates OM is a risk factor for increasing incidence of venous thromboembolism (VTE) development. However, the mechanisms have not been intensively investigated.

**Methods:**

The OM-related dataset GSE30119 and VTE-related datasets GSE19151 and GSE48000 were downloaded from the Gene Expression Omnibus (GEO) database and analyzed to identify the differentially expressed genes (DEGs) (OMGs1 and VTEGs1, respectively). Functional enrichment analyses of Gene Ontology (GO) terms were performed. VTEGs2 and OMGs2 sharing the common GO biological process (GO-BP) ontology between OMGs1 and VTEGs1 were detected. The TRRUST database was used to identify the upstream transcription factors (TFs) that regulate VTEGs2 and OMGs2. The protein-protein interaction (PPI) network between VTEGs2 and OMGs2 was constructed using the Search Tool for the Retrieval of Interacting Genes (STRING) database and then visualized in Cytoscape. Topological properties of the PPI network were calculated by NetworkAnalyzer. The Molecular Complex Detection (MCODE) plugin was utilized to perform module analysis and choose the hub modules of the PPI network.

**Results:**

A total of 587 OMGs1 and 382 VTEGs1 were identified from the related dataset, respectively. GO-BP terms of OMGs1 and shared DGEs1 were mainly enriched in the neutrophil-related immune response process, and the shared GO-BP terms of OMGs1 and VTEGs1 seemed to be focused on cell activation, immune, defense, and inflammatory response to stress or biotic stimulus. 230 VTEGs2, 333 OMGs2, and 13 shared DEGs2 were detected. 3 TF-target gene pairs (SP1-LSP1, SPI1-FCGR1A, and STAT1-FCGR1A) were identified. The PPI network contained 1611 interactions among 467 nodes. The top 10 hub proteins were TP53, IL4, MPO, ELANE, FOS, CD86, HP, SOCS3, ICAM1, and SNRPG. Several core nodes (such as MPO, ELANE, and CAMP) were essential components of the neutrophil extracellular traps (NETs) network.

**Conclusion:**

This is the first data-mining study to explore shared signatures between OM and VTE by the integrated bioinformatic approach, which can help uncover potential biomarkers and therapeutic targets of OM-related VTE.

## 1. Introduction

Venous thromboembolism (VTE), including deep venous thrombosis (DVT) and pulmonary embolism (PE), is a common and severe complication of infectious disease. This complication should always be considered in patients who present with a musculoskeletal infection, especially osteomyelitis (OM) in younger ones [1, 2]. In recent years, the prevalence of DVT among acute haematogenous osteomyelitis (AHO) cases has been reported to be 6–10% [3, 4]. In Taiwan, the risk of developing DVT was 2.49-fold in patients with chronic OM compared with the comparison cohort [5].

Previous studies have evaluated the clinical characteristics of OM among children with and without DVT [3, 4]. *Staphylococcus aureus* (*S. aureus*) is the predominant causative agent for OM [6], and clinical outcomes were worse in patients caused by methicillin-resistant *S. aureus* (MRSA) than in those affected by methicillin-susceptible *S. aureus* (MSSA) [7]. In patients with AHO, Virchow's triad of hypercoagulability, venous stasis, and injury to the vessel wall also applies and is thought to trigger thrombosis. Animal studies have revealed the pathophysiological roles of various influencing factors, including leukocytes, platelets, and neutrophil extracellular traps (NETs), on thrombosis [8]. Besides, there is increasing evidence for an association of Panton–Valentine leukocidin (PVL)-expressing *S. aureus* strains with AHO severity [9]. The possible mechanistic links between DVT and OM may be platelets activated by PVL-damaged neutrophils via neutrophil secretion products [9]. However, the mechanisms by which the transcriptomic response of OM contributes to thrombosis development remain incompletely understood, although there is increasing evidence suggesting an extensive “cross-talk” between the inflammatory and coagulation cascades [10], and these signatures may be useful in the diagnosis of venous thrombosis.

**Figure 1:**
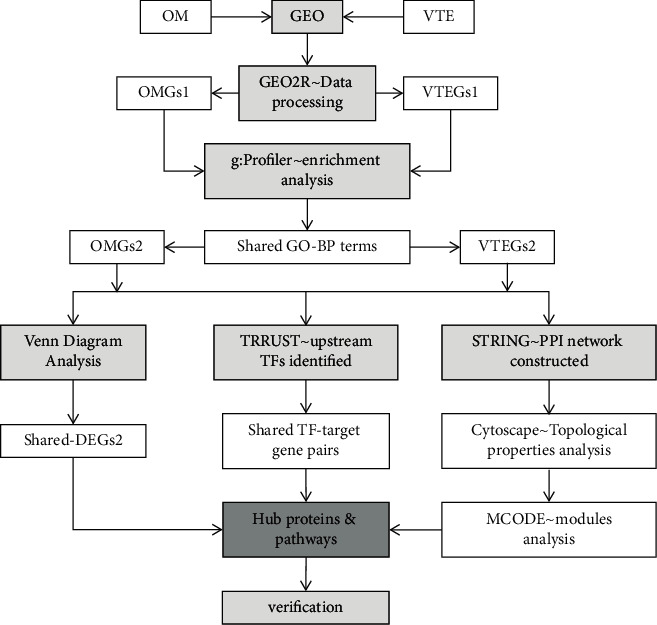
A flowchart of the present study.

Transcriptional profiles are used increasingly to investigate the severity, subtype, and pathogenesis of a disease, which have implications for diagnosis and therapeutic development [11–13]. Therefore, blood transcriptional profiles and host response signatures could serve as biomarkers of clinical changes in subjects at risk for or diagnosed with venous thrombosis in OM. To further explore the underlying pathophysiology, we examined the gene expression alterations involved in OM and venous thrombosis. In this study, we downloaded the gene expression profiles for OM and VTE from the Gene Expression Omnibus (GEO) database. We performed a gene expression analysis using the GEO2R web tool to identify the differentially expressed genes (DEGs) of OM and VTE with their respective controls and subsequently developed Gene Ontology and pathway enrichment analysis for screening of DEGs with the g:Profiler toolset. Finally, an integration of the DEGs protein-protein interaction (PPI) network was constructed and the module analysis was performed. The identified hub genes might play important roles in the process of venous thrombosis which developed from OM. Overall, our findings will hopefully deepen the understanding of the relationships between OM and VTE. A flowchart summarizing this study is shown in Figure 1.

## 2. Materials and Methods

### 2.1. Data Acquisition and Processing

Gene expression datasets related to OM and VTE were obtained from the GEO database of NCBI (https://www.ncbi.nlm.nih.gov/geo/) [14]. The keywords “osteomyelitis” and “venous thromboembolism” with “*Homo sapiens*” or “human” were employed to mine the dataset. Finally, 3 datasets were selected from published studies and downloaded from the GEO database, including GSE30119 for OM and GSE19151 and GSE48000 for VTE. The related information of these datasets is given in Table 1. These blood samples were further divided into different groups according to the diseases source. DEGs were screened using transcription profile data of whole blood samples. All data were normalized and log (base 2) transformed.

### 2.2. Identification of DEGs

According to the recurrence or degree of risk, VTE patients in the two datasets GSE19151 and GSE48000 were grouped and compared with the healthy controls, respectively. The patients with *S. aureus* OM in the dataset GSE30119 were compared with healthy controls. GEO2R web tool (https://www.ncbi.nlm.nih.gov/geo/geo2r/) [18] using the GEOquery and limma *R* packages from the Bioconductor project was utilized to compare the differences in gene expression between two or more groups of samples in a GEO dataset (Table 1) and identify DEGs associated with the diverse experimental conditions. The adjusted *P* values were used to decrease the false positive rate using the Benjamini and Hochberg false discovery rate method by default. The threshold value for identifying DEGs was set as adjusted *P* value < 0.05 and |log2(fold change)| ≥ 1. The results of the pairwise comparisons were summarized for subsequent analysis.

### 2.3. Venn Diagram Analysis of DEGs

We used an online integrative tool Venny (http://bioinfogp.cnb.csic.es/tools/venny/index.html) to analyze the similarities and differences of DEGs in the three datasets, GSE30119, GSE19151, and GSE48000. The identified DEGs of the two datasets associated with VTE were merged, whereas DEGs which were observed in opposite expression directions were discarded. Afterward, two specific clusters of DEGs were defined as OM-related DEGs (OMGs1, obtained from GSE30119) and VTE-related DEGs (VTEGs1, obtained from GSE19151 and GSE48000), respectively. Additionally, the intersections of OMGs1 and VTEGs1 were defined as shared DGEs1.

### 2.4. Functional and Pathway Enrichment Analyses of DEGs

To discern the implication of specific clusters and shared DEGs on OM and VTE, we used the g:Profiler (https://biit.cs.ut.ee/gprofiler/) [19] toolset to perform Gene Oncology (GO) terms and pathway enrichment analysis. The shared biological process ontology (GO-BP) was identified based on overlapping GO term IDs between functional enrichment analysis results of OMGs1 and VTEGs1. The DEGs enclosed by all GO-BP terms shared between OMGs1 and VTEGs1 were defined as DEGs2 (VTEGs2 and OMGs2, respectively) and used to predict the significant pathways and ensuing crosstalk between these pathways. Additionally, the intersections of OMGs2 and VTEGs2, suggested the potential mechanism of patients with OM in whom VTE developed, were defined as shared DGEs2. The Benjamini and Hochberg false discovery rate method was used to correct the *P* value. An adjusted *P* value < 0.05 was considered to have statistical significance and to achieve significant enrichment.

### 2.5. Identification of Transcription Factors (TFs) That Are Significantly Associated with the DEGs

To link gene expression signatures to upstream cell signaling networks, we used Transcriptional Regulatory Relationships Unraveled by Sentence-based Text mining (TRRUST) (https://www.grnpedia.org/trrust/) [20] to identify the upstream TFs that regulate VTEGs2 and OMGs2. The shared TF-target gene pairs of VTEGs2 and OMGs2 may be involved in a common pathway.

### 2.6. PPI Network and Module Analysis

The Search Tool for the Retrieval of Interacting Genes Database (STRING) (https://www.string-db.org/) [21] was used to expose the protein-protein interaction (PPI) information among VTEGs2 and OMGs2 at the protein level. Afterward, the network was recreated and visualized using Cytoscape (http://www.cytoscape.org/) [22], based on the coexpression graph of the PPI network. Topological properties (such as degree of distribution, betweenness centrality, and closeness centrality) of the constructed PPI network were calculated by NetworkAnalyzer in Cytoscape. Nodes with a higher degree based on the number of edges (interactions) between various nodes (proteins) were regarded as hub proteins. The Molecular Complex Detection (MCODE) [23] plugin of Cytoscape was utilized to perform module analysis and choose hub modules of the PPI network with a degree cutoff = 2, node score cutoff = 0.2, k-core ≥ 2, and max. depth = 100.

## 3. Results

### 3.1. Identification of DEGs

**Figure 2:**
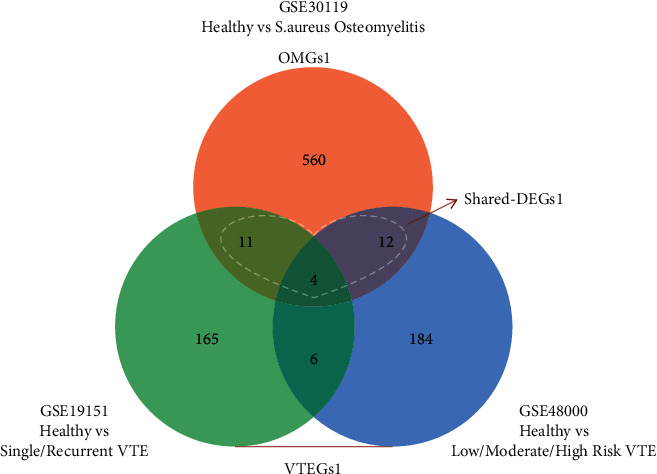
A Venn plot of OMGs1 and VTEGs1. Heart-shaped dotted line represents the shared DGEs1.

**Figure 3:**
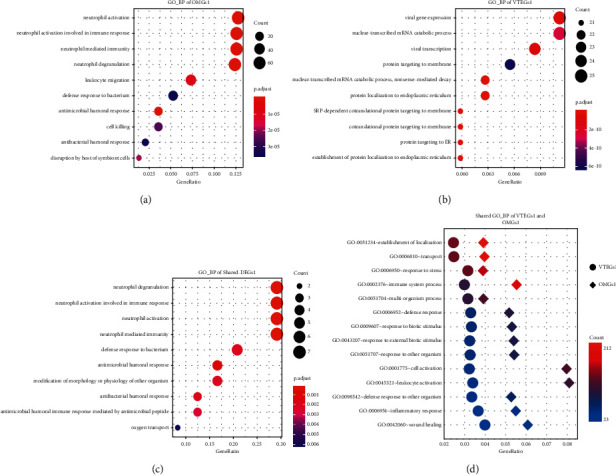
Results of the GO-BP terms enrichment analyses for (a) OMGs1, (b) VTEGs1, and (c) shared DGEs1 and (d) shared GO-BP enrichment of VTEGs1 and OMGs.

**Figure 4:**
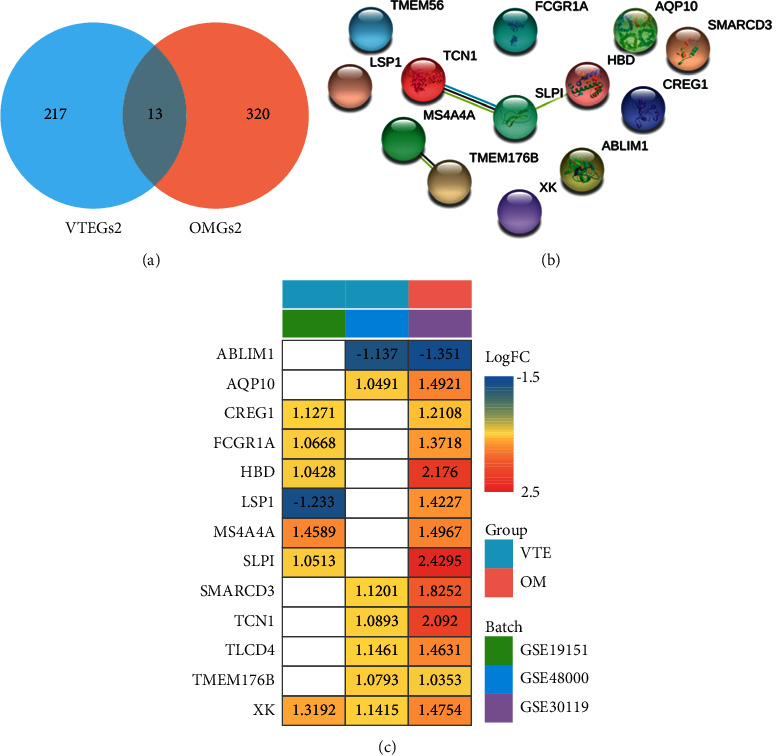
The DEGs2 enclosed by all sharing GO-BP terms. (a) A Venn plot of OMGs2 and VTEGs2. (b) PPI network of the shared DGEs2. (c) Fold change of the shared DGEs2.

Comparisons between differentially gene expression between two or more groups of samples from 1 OM dataset (GSE30119) and 2 VTE datasets (GSE19151 and GSE48000) were performed using the GEO2R web tool, respectively, and mRNAs with an adjusted *P* value < 0.05 and |log2(fold change)| ≥ 1 were identified as DEGs. Two specific clusters of DEGs are shown in Figure 2. A total of 587 OMGs1 were identified in GSE30119. A total of 382 VTEGs1 were identified in VTE-related datasets, including 186 in GSE19151 and 206 in GSE48000. Furthermore, 27 shared DGEs1 that overlap OMGs1 and VTEGs1 were screened out, with 2 genes (LSP1 and PADI4) found to be observed in opposite expression directions. After applying functional annotation with the Gene Ontology database to find terms associated with the development of VTE in OM patients, 230 VTEGs2 and 333 OMGs2 sharing the common biological process ontology were detected and used for further analysis (Figures 3(d) and 4(a)). Afterward, Venn diagram analysis of VTEGs2 and OMGs2 was performed and 13 shared DGEs2 were detected (Figures 4(a)-4(b)). There were 11 upregulated DEGs and 1 downregulated DEG in shared DGEs2, with 1 gene (LSP1) found to be observed in opposite expression directions (Figure 4(c)).

### 3.2. Functional Annotation of DEGs

To gain more biological insight, we performed GO enrichment analysis using the g:Profiler toolset. As shown in Figure 3, the activity of neutrophils has attracted widespread attention among biological processes. With respect to OMGs1, the following GO-BP terms were significantly enriched: neutrophil activation, neutrophil degranulation, neutrophil-mediated immune response, and defense response to the bacterium (Figure 3(a)). Highly similar results of enrichment analysis were also presented in shared DGEs1 (Figure 3(c)). There suggested a significant correlation with the biological process of neutrophils and the process of VTE which developed in OM patients. In addition, VTEGs1 were mainly enriched in the viral gene expression, nuclear-transcribed mRNA catabolic process, protein targeting to membrane, and protein localization to the endoplasmic reticulum (Figure 3(b)). The shared GO-BP terms of OMGs1 and VTEGs1 seemed to be focused on cell activation, immune defense, and inflammatory response to stress or biotic stimuli (Figure 3(d)). These results showed that DEGs were mainly enriched in host immune defense processes such as neutrophil activation and degranulation to resist bacterial invasion or other stress.

### 3.3. Identification of TF-Target Gene Pairs

In the TRRUST database, 36 and 43 upstream TFs were detected to regulate VTEGs2 and OMGs2, respectively. A total of 13 shared upstream TFs were E2F1, GATA1, JUN, NFKB1, RELA, RUNX1, SIRT1, SP1, SPI1, STAT1, STAT3, TP53, and YY1, and these data are given in Table 2. Furthermore, 3 TF-target gene pairs may share a common pathway, consisting of SP1-LSP1, SPI1-FCGR1A, and STAT1-FCGR1A. Interestingly, only one TF-target gene pair has a *P* value less than 0.05.

### 3.4. Hub Proteins and Module Screening of the PPI Network

**Figure 5:**
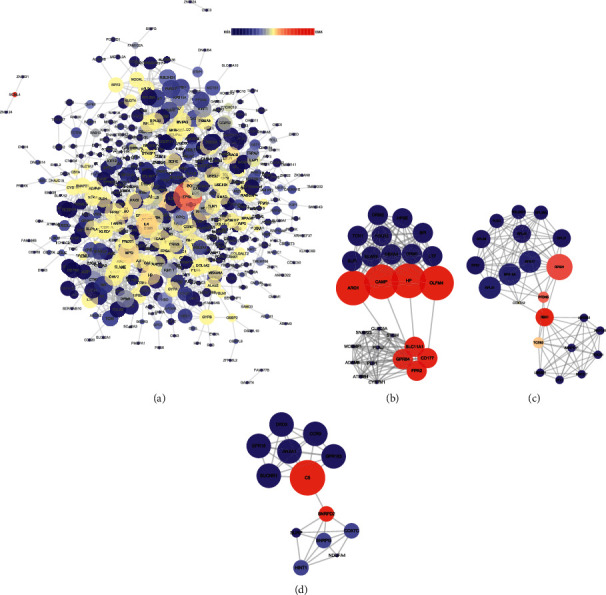
Analysis of the PPI network of DEGs2. (a) PPI network of OMGs2 and VTEGs2. (b) Module 1 of the PPI network of DEGs2. (c) Module 2 of the PPI network of DEGs2. (d) Module 3 of the PPI network of DEGs2. The node sizes correspond to the degree of the node, while the node color denotes betweenness centrality.

A PPI network was utilized to visualize protein-protein interactions involved in the development of VTE in OM patients, and then, potential hub genes between VTEGs2 and OMGs2 were detected. Nodes that did not interact with other nodes were excluded. There were a total of 1611 interactions among 467 nodes (Figure 5(a)). The genes with the highest degree (>30) of interactions within the network were TP53, IL4, MPO, ELANE, FOS, and CD86 (Table 3). To screen significant modules in the PPI network, the MCODE plugin was used in Cytoscape, and the module analysis results are given in Table 4. Top 3 of the 16 modules were illustrated (Figures 5(b), 5(c), 5(d)). The top 5 proteins with higher node degrees in module 1 were ELANE (degree = 32), HP (degree = 29), ORM2 (degree = 25), ARG1 (degree = 25), and CAMP (degree = 24) (Figure 5(b)). The top 5 proteins in module 2 were SOCS3 (degree = 27), RBX1 (degree = 25), UBE2D1 (degree = 24), RPS15A (degree = 22), and RPS17 (degree = 21) (Figure 5(c)). Additionally, the top 5 proteins in module 3 were SNRPG (degree = 26), NDUFA4 (degree = 20), SNRPD2 (degree = 19), COX7C (degree = 19), and HINT1 (degree = 19) (Figure 5(d)). Interestingly, we found that 4 shared DGEs2 were included in these 16 modules, consisting of SLPI (module 1, degree = 17), TCN1 (module 1, degree = 14), CREG1 (module 4, degree = 11), and FCGR1A (module 16, degree = 19) (Table 4).

## 4. Discussion

As a fatal disease caused by serious musculoskeletal infection, VTE needs to arouse the attention of clinical staff. It has been reported that children with OM may have increased susceptibility to VTE in previous epidemiologic studies [3–5]. Unfortunately, the transcriptional signatures between VTE and OM have not been intensively investigated. The main objective of this study was to focus on revealing the underlying pathophysiology association between VTE and OM.

In the present study, we integrated 2 expression profiles from VTE patients and 1 expression profile from OM patients to identify genes that may play a crucial role in the onset and development of VTE in OM patients. First, 382 VTEGs1 and 587 OMGs1 were identified in VTE and OM patients, respectively. Following these, DEGs1 were evaluated by functional enrichment analysis to get insight into the biological significance in the pathogenesis. It is reported that blood coagulation was overexpressed in patients with osteoarticular infections [15, 24]. Here, our research showed that OMGs1 and shared DGEs1 were mainly enriched in some neutrophils-related pathways, such as neutrophil activation, neutrophil degranulation, neutrophil-mediated immune response, and defense response to the bacterium. Moreover, the shared GO-BP terms of OMGs1 and VTEGs1 seemed to be focused on cell activation, immune defense, and inflammatory response to stress or biotic stimuli. These results indicated that the activity of neutrophils and the immune response to bacterial invasion may explain the molecular mechanisms of VTE developed in OM patients to some extent. After that, 230 VTEGs2 and 333 OMGs2 sharing the common biological process ontology were detected by comparing GO-BP terms of VTEGs1 and OMGs1. Additionally, 13 shared DGEs2 that overlap OMGs2 and VTEGs2 were identified for further analysis.

It is well known that TFs, specific to binding to its target gene, exert facilitative or inhibitory roles in gene expression, showing an important part in the multitude of biological processes involved in diseases [20]. In this study, based on the Venn diagram analysis method, 13 shared upstream TFs of VTEGs2 and OMGs2 were screened out. It is suggested that 3 TF-target gene pairs, consisting of SP1-LSP1, SPI1-FCGR1A, and STAT1-FCGR1A, may reveal the potential pathogenesis link between VTE and OM. Finally, in the PPI network analysis, TP53, IL4, MPO, ELANE, FOS, CD86, HP, SOCS3, ICAM1, and SNRPG were the top 10 hub proteins with the highest connectivity within the network. Significant modules were further detected in the PPI network, and some specific nodes with the highest connectivity (i.e., ELANE, HP, SOCS3, and SNRPG) were also identified in the top 3 modules. Particularly, 4 shared DGEs2 (SLPI, TCN1, CREG1, and FCGR1A) were found in the 16 modules.

OM, especially haematogenous OM, can cause an excessive inflammatory response in blood vessels and coagulation disorders modulated by various microorganisms [25, 26]. *S. aureus* is the predominant causative pathogen of OM [27]. Surface proteins and exotoxins of *S. aureus* can trigger thrombosis through effects on the coagulation pathway and on anticoagulation factors. Additionally, *S. aureus* can activate endothelial surfaces and platelets. It has been reported that some exotoxins such as PVL expressed by *S. aureus* can contribute to leukocyte lysis and additional damage to endothelial surfaces [9, 28]. During *S. aureus* infection, platelets amplify the recruitment and activation of innate immune cells at the site of infection and help eliminate pathogens. In some cases, these mechanisms can lead to thromboinflammation, leading to severe organ dysfunction. These events can lead to microthrombosis and DVT. Neutrophils act as the first line of defense in the process of acute infection. They can protect the human body from various pathogens through antibacterial mechanisms such as phagocytosis, degranulation, and the production of reactive oxygen species (ROS). According to recent research, neutrophils can also exert their innate immune function by forming a special structure called NETs [29–31]. Our previous study also suggested that NETs may play a crucial role in the immune response of patients with OM during the *S. aureus* infection process [24]. In this study, some core nodes (such as MPO, ELANE, and CAMP) were essential components of the NETs network structure. Emerging research focused on how NETs affect platelet function, particularly in the setting of infection and inflammation [32–34]. In patients with sepsis, systemic inflammation primed neutrophils to release NETs, which promoted thrombin generation through platelet-dependent and platelet-independent mechanisms, with the increased risk of VTE [35, 36]. There may also be a similar underlying mechanism between *S. aureus* OM and the development of DVT in the extremities. In addition, early markers detection and targeted intervention may minimize this effect in the inflammatory process of thrombosis in patients with OM.

Nowadays, TFs are becoming attractive biomarkers in complex diseases including infectious diseases. Among the identified regulatory TFs, Sp1 participated in the hypoxia-induced upregulation of VWF [37]. Increasing evidence indicates that the interaction between NETs and VWF contributes to arterial and venous thrombosis as well as inflammation [34]. As a target gene of SP1, endothelial LSP1 is recruited into the cytoskeleton during inflammation and plays an important role in the formation of endothelial dome, thereby regulating the transendothelial migration of neutrophils [38]. The haematopoietic transcription factor SPI1/PU.1 may be specifically involved in the differentiation or activation of macrophages or B cells. Moreover, SPI1 was able to restrain neutrophil defense by broadly inhibiting the accessibility of enhancers via the recruitment of histone deacetylase 1 [39]. STAT1 can be phosphorylated by receptor-associated kinases and then shapes' cell viability in response to different cell stimuli and pathogens. The activation of STAT1 inhibits angiogenesis [40], which may be involved in the inflammatory process of thrombosis in infectious diseases. FCGR1A is the common downstream target gene of SPI1 and STAT1. FCGR1A, also known as CD64, as an important immune-related protein, is closely related to various inflammatory processes. In posttraumatic OM patients, immunocompetent cells, predominantly highly activated polymorphonuclear neutrophils, as characterized by low expression of CD62L and high expression of the adhesion protein CD18, of the high-affinity IgG receptor CD64 and of the LPS receptor CD14, were recruited into the site of infection [41].

Additionally, other hub proteins may also play a crucial role in the pathogenesis of VTE in patients with OM. TP53, the top hub gene of our study, is a critical tumor suppressor and a key regulator in numerous cellular functions, which maintains critical functions in immunity, inflammation, and tissue repair [42]. Intriguingly, TP53 may participate in the host-virus interactions that could characterize shared biological mechanisms between acute respiratory distress syndrome (ARDS) and VTE in severe COVID-19 patients [43]. A similar host-bacterial interaction may also be reflected between OM and VTE. IL4 is directly involved in the bone desorption and osteoclast activity regulation that occur in OM. The association between IL4 gene polymorphisms (-1098-G/T and -590-C/T) and chronic OM was previously observed [44]. However, IL4 was not significantly elevated in a mouse model of fracture fixation with *S. aureus* OM [45]. The possible links between inflammation-related genetic variants (including IL4) and VTE established the fundamental role of genetic background in predisposition to VTE and several inflammation-related conditions [46]. HP, one of the acute-phase reactants, can stimulate inflammatory bone loss and its phenotype 2-2 has been reported to be a risk factor for spontaneous VTE [47]. SOCS3 was reported to be a key player in bone-associated inflammatory responses, which acted as the cytokine-inducible negative regulator of cytokine signaling [48]. The FOS proteins have been implicated as regulators of cell proliferation, differentiation, and transformation. The c-Fos gene has been put forward as a new factor in the progression and severity of atherosclerosis [49]. CD86 plays an important role in immune responses as a costimulatory molecule on antigen presenting cells. The gene polymorphisms of CD86 may be related to the risk of sepsis with contradictory results in different studies [50, 51]. Unfortunately, there is no relevant research report on FOS or CD86 in the pathogenesis of OM or VTE.

This study has several limitations. Although followed the same analysis strategy and threshold for DEGs screening, the number of differential genes shared by the GSE19151 and GSE48000 datasets obtained in the end was small due to the inconsistency of the inclusion criteria and sequencing platform. The DEGs from these two datasets were included in the subsequent analysis, which can provide us with more possibilities, but they were not differentially expressed at the same time. More external datasets were needed to verify the results of the study. In addition, further experimental investigations are warranted to decipher the roles of these hub genes in the development of VTE in OM patients.

## 5. Conclusions

In this study, we reported for the first time the identification of the DEGs and activated signaling pathways between OM and VTE by the integrated bioinformatic approach. Our results finally identified a new set of novel biomarkers and important molecular targets, including 3 TF-target gene pairs (SP1-LSP1, SPI1-FCGR1A, and STAT1-FCGR1A), 3 structural proteins of NETs (MPO, ELANE, and CAMP), and 6 other hub proteins (TP53, IL4, HP, SOCS3, FOS, and CD86), which might play essential biological roles during the progression of VTE in OM patients. In addition, the enriched neutrophils-related pathways (neutrophil activation, degranulation, and immune response to bacteria) could advance our understanding of the development of OM towards VTE and indicate new avenues to develop therapies for VTE. However, more research, especially experimental research, will be indispensable for the further clinical application of these biomarkers.

## Figures and Tables

**Table 1:** Published datasets related to OM and VTE used in this study.

Accession	Platform	No. of control	No. of disease	Sample source	Reference
GSE30119	GPL6947: Illumina HumanHT-12 V3.0 Expression BeadChip	44 healthy	57 osteomyelitis (4 emboli, 4 DVT)	Whole blood	[15]
GSE19151	GPL571: [HG-U133A_2] Affymetrix Human Genome U133A 2.0 Array	63 healthy	32 single VTE; 38 recurrent VTE	Whole blood	[16]
GSE48000	GPL10558: Illumina HumanHT-12 V4.0 Expression BeadChip	25 healthy	40 high-risk VTE; 33 moderate-risk VTE; 34 low-risk VTE	Whole blood	[17]

**Table 2:** The list of TF-target gene pairs of VTEGs2 and OMGs2.

Shared TFs	VTEGs2				OMGs2			
	No.	Target genes	*P* value	Q value	No.	Target genes	*P* value	Q value
E2F1	2	TP53, NRIP1	0.483	0.497	5	CDKN1C, PPARG, RRM2, IL23A, PCSK6	0.0863	0.124
GATA1	4	MPL, DICER1, HBE1, GYPB	0.005	0.0245	3	PRG2, ALAS2, FCER1A	0.0778	0.115
JUN	3	CSTA, NAMPT, TP53	0.269	0.312	3	IL23A, CTGF, PLAU	0.485	0.496
NFKB1	5	CD3G, NAMPT, BCL2A1, ICAM1, TP53	0.304	0.332	12	PTGES, TFF3, COL1A2, SOCS3, PLAU, HOXA9, CR2, HPSE, CD86, IL4, OLFM4, IL23A	0.00727	0.0347
RELA	5	BCL2A1, TRIB3, ICAM1, NAMPT, TP53	0.299	0.332	11	TFF3, COL1A2, IL4, IL23A, CD86, PTGES, PLAU, OLFM4, HPSE, CR2, SOCS3	0.0172	0.0674
RUNX1	2	PIK3CD, MPL	0.0841	0.121	3	MPO, ELANE, BPI	0.0325	0.0874
SIRT1	3	HBE1, ICAM1, TP53	0.0202	0.0519	2	PPARG, COL1A2	0.205	0.242
SP1	9	CYP1B1, ICAM1, LSP1, FBLN1, CD151, ATP2A3, MCTS1, DCK, FOS	0.119	0.153	9	MMRN1, MPO, LSP1, MME, CTGF, ITGB3, PLAU, PTGES, LTF	0.443	0.464
SPI1	3	FCGR1A, ACP5, BCL6	0.0391	0.0741	8	FCER1A, ELANE, BPI, P2RY10, CTSG, FCGR1A, PRG2, MME	1.19E-05	0.000512
STAT1	3	TP53, ICAM1, FCGR1A	0.0815	0.121	4	CD86, FCGR1A, IL1R1, SOCS3	0.0597	0.112
STAT3	4	FOS, ICAM1, BCL6, TP53	0.0936	0.13	4	PROS1, CTGF, HP, SOCS3	0.237	0.262
TP53	2	TP53, PMAIP1	0.592	0.592	3	PAX5, NLRC4, VCAN	0.549	0.549
YY1	3	TP53, COX7C, HBE1	0.0979	0.131	4	COL1A2, DNAJB4, FCER1A, IL4	0.0754	0.115

**Table 3:** The top 30 hub proteins identified in the topological analysis of the PPI network of DEGs2.

No.	Gene	Degree	Betweenness centrality	Closeness centrality
1	TP53	74	0.34740282	0.43589744
2	IL4	39	0.0760529	0.38442211
3	MPO	38	0.05899592	0.37996689
4	ELANE	32	0.02590356	0.35253456
5	FOS	31	0.0748293	0.38059701
6	CD86	31	0.03757972	0.34330591
7	HP	29	0.04561475	0.36
8	SOCS3	27	0.03327054	0.36113297
9	ICAM1	27	0.02949815	0.37135922
10	SNRPG	26	0.02319152	0.29105897
11	ORM2	25	0.00924872	0.30722892
12	RBX1	25	0.02116545	0.33874539
13	ARG1	25	0.01228008	0.32438163
14	FPR2	24	0.0214619	0.31808732
15	UBE2D1	24	0.02571099	0.33675715
16	CAMP	24	0.00896381	0.32233146
17	BPI	23	0.00389229	0.29708738
18	RPS15A	22	0.00569474	0.28456293
19	DEFA4	22	0.00259991	0.29708738
20	RPS17	21	0.004417	0.28125
21	RPS21	21	0.00432586	0.28039096
22	RPL31	21	0.00408646	0.28021978
23	MAPK14	21	0.04653847	0.3538936
24	CTSG	21	0.0063411	0.29708738
25	CD79A	21	0.01986526	0.34253731
26	PLAU	21	0.04559923	0.35334873
27	RPL39	20	0.01096077	0.29254302
28	ORM1	20	0.00663223	0.29404228
29	NDUFA4	20	0.02048315	0.29479769
30	LTF	20	0.00846729	0.29517685

**Table 4:** The module analysis results with MCODE of the PPI network of DEGs2.

Module	Score (density^*∗*^#nodes)	Nodes	Edges	Node IDs
1	13.308	27	173	CYSTM1, PLAU, LTF, ADAM8, TCN1, DEFA4, OLFM4, CD177, BPI, STOM, GPR84, CLEC5A, FPR2, CAMP, PLD1, HP, MCEMP1, ORM2, ARG1, ORM1, HPSE, FOLR3, SNAP23, ELANE, SLPI, SLC11A1, ATP8B4
2	10.19	22	107	RBX1, PLEC, COX7A2, UBE2D1, RPS17, RPS15A, RPL23, RPL36A, UFL1, RPS7, RSL24D1, RPL39, RPL21, RPS21, UBE2D2, ANAPC11, RPL31, RNF217, PFDN5, SOCS3, TCEB2, RNF130
3	5.833	13	35	GPR183, GPR18, COX7C, CCR9, ANXA1, C5, DRD3, SLIRP, HINT1, NDUFA4, SNRPD2, SNRPG, SUCNR1
4	5.5	9	22	S100A12, CTSA, DEFA1B, SDCBP, MPO, CREG1, DEFA1, DEFA3, RNASE3
5	5	5	10	GAS6, PROS1, SERPING1, MMRN1, F5
6	4.471	18	38	VCAN, IL1R1, HIST2H2AC, BCL6, IGFBP7, CD79A, MME, FOS, ICAM1, HIST2H2AA3, PDLIM7, FSTL1, MAPK14, PPARG, CR2, PRSS23, PAX5, BARD1
7	4	4	6	CEP83, TCTN3, TMEM67, ODF2
8	4	4	6	CD86, HLA-DQB1, CD3G, CD3D
9	3.333	4	5	NDUFA5, UQCR11, UQCRB, C14orf2
10	3.182	23	35	CACNA1E, COL17A1, CACNB2, COLGALT2, C1QB, GRAP2, MYL4, SNCA, GAB2, HBE1, P2RY10, HMBS, AHSP, MB, RASGRP4, GYPB, SIGLEC1, ALAS2, NTSR1, PLOD2, PIK3CD, IL4, CACNG6
11	3	3	3	LSR, PEX3, APOBR
12	3	3	3	KLRF1, KIR2DL1, KIR3DL2
13	3	3	3	MRPL48, GADD45GIP1, MRPS31
14	3	3	3	STRBP, THAP4, ZCCHC10
15	3	3	3	IRX3, HOXA9, HOXA10
16	2.857	15	20	IL6ST, TRIM17, IL23A, H2AFJ, HIST1H1C, ANPEP, CALD1, TP53, TRAT1, FCGR1B, ITGB3, FCGR1A, HIST2H3C, COL1A2, STAT6

## Data Availability

The data used to support the findings of this study are available from the corresponding author upon request.
